# Central retinal artery occlusion associated with patent foramen ovale: a case report and literature review

**DOI:** 10.5935/0004-2749.20210073

**Published:** 2021

**Authors:** Matthew S. Wieder, Nancy Blace, Moshe M. Szlechter, Eric Shulman, Jincy Thankenchen, Joyce N. Mbekeani

**Affiliations:** 1 Department of Ophthalmology & Visual Sciences, Montefiore Medical Center/AECOM, New York, USA; 2 Department of Ophthalmology, Bronx Lebanon Hospital Center of Icahn School of Medicine at Mt Sinai, New York, USA; 3 Department of Surgery (Ophthalmology), Jacobi Medical Center, New York, USA; 4 KLM Eye MDs, Brooklyn, New York, USA; 5 Department of Medicine (Cardiology), Jacobi Medical Center, New York, USA

**Keywords:** Retinal artery occlusion, Foramen ovale, patent, Transesophageal, Echocardiography, Case reports, Oclusão da artéria retiniana, Forame oval patente, Ecocardiografia transesofágica, Ecocardiografia, Relatos de casos

## Abstract

Patent foramen ovale might cause cryptogenic strokes, including retinal artery occlusion. Herein, we describe a previously healthy young man who presented with central retinal artery occlusion in the setting of patent foramen ovale and explore the need for transesophageal echocardiogram for its diagnosis. Cardiovascular workup and neuroimaging were unremarkable. Transthoracic echocardiogram bubble study revealed a right to left atrial shunt and subsequent transesophageal echocardiogram disclosed patent foramen ovale. This congenital cardiac anomaly was the likely conduit for a thrombo-embolic central retinal artery occlusion. We identified seven patients with patent foramen ovale associated with central retinal artery occlusion in the literature. Transthoracic echocardiogram was diagnostic in only one patient (14.3%), whereas transesophageal echocardiogram was required to reveal patent foramen ovale in the remaining six (85.7%). Our case and the previous reports support the link between central retinal artery occlusion and patent foramen ovale. Therefore, providers should consider the more sensitive transesophageal echocardiogram during the initial evaluation of young patients without immediately identifiable causes of retinal artery occlusion.

## Abstract (pt)

O forame oval patente pode estar associado a derrames criptogênicos que incluem a oclusão da artéria retiniana. Descrevemos aqui um jovem previamente saudável que apresentou oclusão da artéria central da retina associada ao forame oval patente, sendo considerado portanto, a necessidade de um ecocardiograma transesofágico para seu diagnóstico. A avaliação cardiovascular e a neuroimagem não foram significativas. O estudo da bolha no ecocardiograma transtorácico revelou um shunt atrial direito-esquerdo e o ecocardiograma transesofágico subsequente revelou um forame oval patente. Esta anomalia cardíaca congênita foi o provável conduíte para uma oclusão tromboembólica da artéria central retiniana Na literatura, foram identificadossete pacientes com forame oval patente associado à oclusão da artéria central retiniana. O ecocardiograma transtorácico diagnosticou apenas um paciente (14,3%), enquanto o ecocardiograma transesofágico foi necessário para revelar o forame oval patente nos seis casos restantes (85,7%). Nosso caso e relatos anteriores suportam a ligação entre a oclusão da artéria central retiniana e o forame oval patente. Os profissionais devem considerar, como sendo mais sensível, o ecocardiograma transesofágico na avaliação inicial de pacientes jovens sem causas imediatamente identificáveis de oclusões da artéria retiniana.

## INTRODUCTION

Patent foramen ovale (PFO) is a connection between the right and left atria. During fetal development, presence of a PFO allow oxygenated maternal blood to bypass lung circulation and directly supply the arterial circulation. In most, the PFO closes spontaneously during infancy^(1)^. However, in 27% of the general population, this fetal shunt persists. In the absence of filtration in the lungs, emboli emanating from silent deep or superficial venous thrombosis traverse this shunt causing paradoxical emboli. The risk of stroke in those without comorbidities is only 0.1%^(1)^. However, in patients under 55 years, up to 46% of cryptogenic strokes have been attributed to PFO^(1)^.

Central retinal artery occlusion (CRAO), a stroke of the inner retina, presents as acute painless vision loss, typically resulting in 20/400 vison or worse^(2)^. It has an estimated incidence of 1 in 100,000^(2)^, and has a mean age of presentation of 60 years^(3)^. The common etiolo gies include cardiac abnormalities, coagulopathies, myeloproliferative disorders, collagen vascular diseases, as well as other inflammatory diseases and malignancy. In approximately 45% of patients under 45 years of age, CRAO is associated with underlying cardiac abnormalities^(4)^. Besides profound vision loss affecting the functional capacity, patients are at increased risk of cerebral and cardiac ischemia, warranting evaluation of underlying etiologies.

Transthoracic echocardiography (TTE) is part of standard stroke workup to exclude cardiac etiology. It employs a non-invasive, ultrasound probe or transducer applied to the chest. Ultrasound waves are translated into video images to assess cardiac anatomy and physiology. Furthermore, bubble accentuated studies using intravenous injected agitated saline (gas added to saline) assess the cardiac flow. Even though this technique can reveal shunts, it cannot differentiate atrial septal defects from PFO.

Transesophageal echocardiogram (TEE) has been used to supplement TTE. A flexible probe with the ultra sound transducer at the tip is inserted into the esophagus, placing it more proximal to the heart. The signal-weakening effect of intervening thoracic structures is reduced, improving the resolution of cardiac images. Therefore, PFOs are easily visualized and quantified. Previous studies have determined that PFO is one of the most common cardiac defects determined using TEE in patients with cryptogenic embolic strokes, with PFO being detected after the initial TTE reported no abnormal findings^(5)^. Herein, we report an illustrative case of CRAO associated with PFO. We reviewed the English literature for similar cases and explored the types of echocardiograms required to disclose this cardiac anomaly.

## CASE REPORT

**Figure 1:**
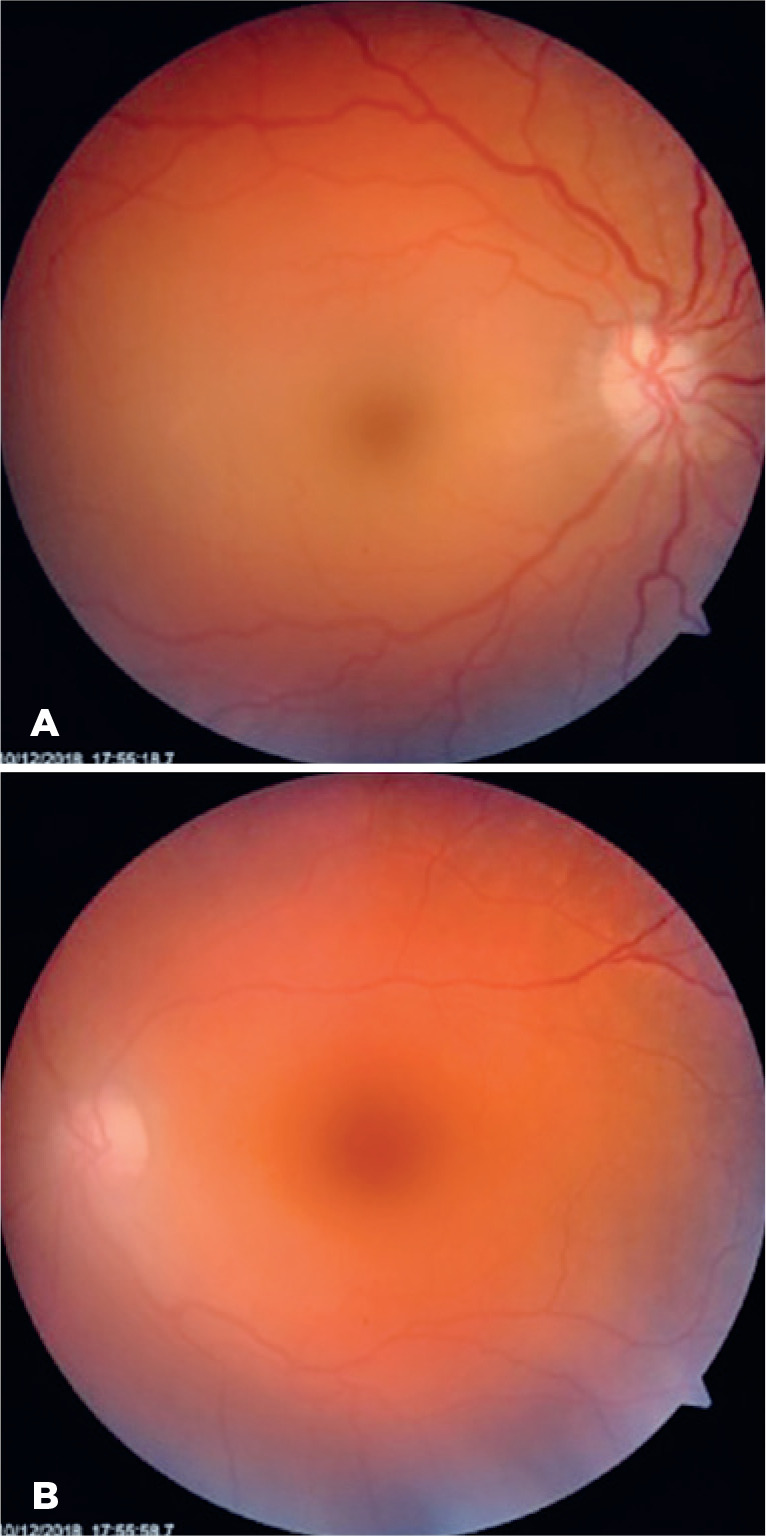
Color images of fundus in a young man with right central retinal artery occlusion. A) Fundus image of the right eye demonstrating nerve pallor with mild retinal edema and pallor and central cherry red spot. B) Fundus image of the normal left eye.

**Figure 2:**
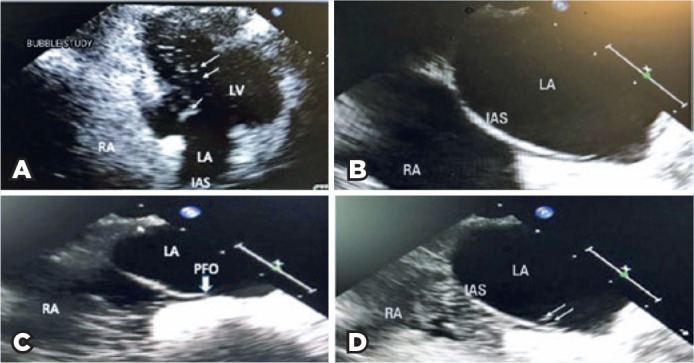
Echocardiographic images of a young patient with right central retinal artery occlusion. A) Transthoracic echo (TTE) image illustrating a moderate amount of agitated saline bubbles appearing from the left atrium to the left ventricle suggesting a right to left shunt. Arrows point to agitated saline. This finding revealed presence of a right to left shunt and led to further imaging with a transesophageal echo to assess the shunt further. B) Transesophageal echocardiogram (TEE) image of interatrial septum at baseline. C) Transesophageal echocardiogramgrevealing a patent foramen ovale (PFO) with agitated saline passing through from the right atrium and into the left atrium. Arrow indicates PFO and agitated saline passing through PFO. D) Transesophageal echocardiogram confirms PFO as the interatrial shunt. There are agitated saline bubbles seen in both right and left atrium (arrows).

A 43-year-old male with a history of hypertension presented with sudden painless right vision loss. Examination revealed best-corrected visual acuities of no light perception (right) and 20/25 (left) and right relative afferent pupillary defect. Intraocular pressures were normal, extraocular movements were full, and anterior segments were unremarkable. Dilated fundus examination revealed a right pale, moderately swollen optic nerve, macular pallor with a cherry red spot, and box-carring blood flow in several vessels. However, emboli were not detected. The left eye was normal. Right central retinal artery occlusion was diagnosed, and the patient was transferred to the Stroke Unit for admission and management. Blood pressure on admission was 136/92 mmHg. Exa mination the next day revealed resolution of box-carring and a more distinct cherry red spot (Figure 1A). The left eye remained normal (Figure 1B). Fundus fluo rescein angiogram (FFA) revealed no abnormalities and no evidence of emboli, arterial narrowing, staining, or areas of non-perfusion. Laboratory workup revealed elevated total cholesterol of 220 mg/dL (normal= 120200mg/dL): high density lipoprotein of 33 mg/dL (normal = 27-67 mg/dL), low density lipoprotein of 154.6 mg/dL (normal = 100-129 mg/dL), and triglycerides of 162 mg/dL (normal = 40-160 mg/dL). Complete blood count, including platelet count, erythrocyte sedimentation rate (ESR), C-reactive protein (CRP), coagulation profile (PT/PTT), glycosylated hemoglobin (HbA1c), urine toxicology, anti-cardiolipin, lupus anticoagulant, antinuclear antibodies (ANA), antineutrophil cytoplasmic antibodies (ANCA), homocysteine levels, angiotensin converting antibody (ACE), syphilis antibodies, and C and S proteins were normal. Head and neck magnetic resonance imaging and magnetic resonance angiography (MRI/ MRA) were normal, and carotid duplex was negative for stenosis or occlusions. Holter monitor did not reveal any arrhythmia and venograms failed to disclose venous thrombosis. TTE (S5 transducer, Philips iE33 model, *Koninklijke Philips N.V., Amsterdam, Netherlands*) was initially normal; however, a subsequent bubble study revealed an interatrial right to left shunt (Figure 2A). TEE (X5 transducer, Philips iE33 model) performed to further define the shunt revealed a small interatrial tunnel, characteristic of PFO (Figures 2B-D).

R= right atrium; L= left atrium; IAS= interatrial septum; L= left ventricle).

The patient’s hospital course was uneventful, and he was discharged on lisinopril, nifedipine, aspirin, and atorvastatin. At the one-month follow-up, right visual acuity improved to count fingers and fundus examination revealed resolution of retinal pallor without evidence of neovascularization. A silent venous thrombo-emboli traversing the PFO was thought to be causally related to the CRAO. The patient was referred to another center for consideration of PFO closure to reduce the risk of additional ischemic vasculo-occlusive events.

## DISCUSSION

Our case demonstrated a young patient presenting with CRAO with initially negative cardiovascular and neurologic workup. Although initial TTE bubble study revealed a right to left atrial shunt, it was not diagnostic for PFO. TEE was diagnostic for PFO and verified the location and size. This observation has implications for pursuing TEE in young patients after cryptogenic vascular events. Even though our patient had a history of hypertension and investigations disclosed hyperlipidemia that could have contributed to CRAO, both conditions were deemed mild and likely noncontributory. Hence, the PFO was considered to be the likely cause of CRAO.

Our review of published cases of concurrent CRAO and PFO yielded seven reports (Table 1)^(3,6-10)^. The mean (SD) ages were 42.4 years (27.1 years). Relevant histories included hypertension and smoking. TTE was positive in only one patient (14.3%). In this case, TTE required augmentation with contrast to disclose the PFO^(3)^. TEE was performed on six patients (85.7%), revealing PFO. All these patients had preceding TTE that had failed to disclose PFO^(6-10)^. In their study, Inatomi et al.^(6)^ evaluated 22 consecutive patients with retinal artery occlusion and observed that 59% had cardiac abnormalities detected using TEE compared with only 27% detected using TTE.

**Table 1:** Summary of Previous Reports of Retinal Artery Occlusion and Patent Foramen Oval

Article	Type of Age/ retinal artery sex occlusion		Side	Visual acuity	Medical problems	Fundus findings	TTE	PFO on TTE	TEE	PFO on TEE Other imaging	Labs	Treatment
Clifford et al.<^^3^^>	22/M	CRAO	OS	NLP	Smoker	RAPD, attenuation, swollen disc, central retinal embolus	Y	+ ^*^		B-scan, MR1, ECG, CD all WNL	Mildly elevated homocysteine level otherwise all hematological and biochemical, autoimmune, prothrombotic and infections all negative	Aspirin, folic acid. Planned for percutaneous closure
Inatomi et al.^(6)^	78/F	CRAO	NS	NS	HTN	NS	Y	-	Y	+ NS	NS	NS
Ho et al.^(7)^	15/M	CRAO^r^	OS	20/60	Fractured clavicle	RAPD, inner retinal ischemic whitening with fovea sparing	Y	-	Y	+ Carotid Doppler, otherwise, NS	NS	Referred for surgery
Gabrielian et al.^(8)^	17/M	CRAO	OD	HM	none	Cherry red spot	Y	-	Y	+ NS	Hematological and infectious workup negative.	Percutaneous closure
Nakagawa et al.^(9)^	43/F	CRAO	OU	4/200^Ω^	HTN	Cherry red spot and delayed AV transit time on FFA	Y		Y	+ MRl/MRA, CD	Routine blood work as well as ANA, anti dsDNA, lupus anticoagulant, anticardiolipin, antithrombin 111, alpha 2 plasmin inhibitor, protein S and protein C	Anticoagulantnot specified
Hayashi et al.^(10)^	79/F	CRAO	OS	LP	HTN	Delayed arterial filling	Y		Y	+ Transcranial Doppler - right to left shunt. MR1 -hyperintense lesion in the left MCA. Cerebral angiography-mild atherosclerotic changes in the left dlCA without atherosclerotic changes in the ophthalmic artery or plCA. LE dopplerleft peroneal vein with massive thrombus, ECG -WNL	D-dimer and antithrombin 111 elevated	Warfarin
Wieder et al (this report)	43/M	CRAO	OD	NLP	HTN	RAPD, pale optic nerve with a cherry red spot and diffuse box-carring blood flow in retinal vessels	Y		Y	+ MRl/MRA, CD, Holter monitor	Elevated cholesterol, CBC, PT, PTT, HbA1c, urine toxicology, ESR, CRP, anti-cardiolipin, ANCA, ANA, homocysteine, VDRL, ACE, Protein C/S, lupus anticoagulant all negative	Aspirin and statin. Referred for closure of persistent foramen ovale (PFO)

Γ= cilioretinal sparing; ∆= cilioretinal artery occlusion Ω - Started OD then 6 days later presented HM OS

* = positive on contrast echo only.

PFO= patent foramen ovale, TEE= transesophageal echocardiogram; TTE= transthoracic echocardiogram; CRAO= central retinal artery occlusion; RAPD= relative afferent pupillary defect; OD= right eye; OS= left eye; FHx= family history; NLP= no light perception; HM= hand motion vision; LP= light perception; VF= visual field; FFA= fundus fluorescein angiogram; HTN= hypertension; CD= carotid Doppler; dICA= distal internal carotid artery; pICA= proximal internal carotid artery; LE= lower extremity; MRI= magnetic resonance imaging; MRA= magnetic resonance angiogram; MCA= middle cerebral artery; CBC= complete blood count; ESR= erythrocyte sedimentation rate; CRP= C-reactive protein; HbA1_C_= hemoglobulin A1_C_ (glycosylated hemoglobulin); PT= prothrombin time; PTT= partial thromboplastin time; ANA= anti-nuclear antigen; dsDNA= double-stranded DNA; RF= rheumatoid factor; ANCA -antineutrophil cytoplasmic antibodies; ACE= angiotensin converting enzyme; VDRL= venereal disease research laboratory; SPEP= serum protein electrophoresis; NS= not specified; Y= yes.

Although a prospective, randomized, controlled study with uniform investigations and standardized instrumentation would provide better evidence-based support for the initial investigative protocols of young patients presenting with retinal artery occlusions, our case and the previous reports affirm that TEE has a higher diagnostic yield for PFO than TTE (Table 1). Therefore, providers should consider TEE during the initial workup of young patients with cryptogenic CRAO.
